# Antioxidant and Antibacterial Activity of Essential Oils and Hydroethanolic Extracts of Greek Oregano (*O*. *vulgare* L. subsp. *hirtum* (Link) Ietswaart) and Common Oregano (*O*. *vulgare* L. subsp. *vulgare*)

**DOI:** 10.3390/molecules26040988

**Published:** 2021-02-13

**Authors:** Olga Kosakowska, Zenon Węglarz, Ewelina Pióro-Jabrucka, Jarosław L. Przybył, Karolina Kraśniewska, Małgorzata Gniewosz, Katarzyna Bączek

**Affiliations:** 1 Department of Vegetable and Medicinal Plants, Institute of Horticultural Sciences, Warsaw University of Life Sciences-SGGW, Nowoursynowska 166, 02-787 Warsaw, Poland; zenon_weglarz@sggw.edu.pl (Z.W.); ewelina_pioro_jabrucka@sggw.edu.pl (E.P.-J.); jaroslaw_przybyl@sggw.edu.pl (J.L.P.); katarzyna_baczek@sggw.edu.pl (K.B.); 2 Department of Food Biotechnology and Microbiology, Institute of Food Sciences, Warsaw University of Life Sciences-SGGW, Nowoursynowska 159, 02-776 Warsaw, Poland; karolina_krasniewska@sggw.edu.pl (K.K.); malgorzata_gniewosz@sggw.edu.pl (M.G.)

**Keywords:** essential oils, hydroethanolic extracts, Greek oregano, common oregano, antioxidant power, antibacterial activity

## Abstract

Greek oregano and common oregano were compared in respect of the antioxidant and antibacterial activity of the corresponding essential oils and hydroethanolic extracts in relation with their chemical profile. The chemical composition of essential oils was determined by GC-MS and GC-FID, while extracts (phenolic acids and flavonoids fractions) were analyzed by HPLC-DAD. Based on given volatiles, the investigated subspecies represented two chemotypes: a carvacrol/γ-terpinene/*p*-cymene type in the case of Greek oregano and a sabinyl/cymyl type rich in terpinen-4-ol in common oregano. Within non-volatile phenolic compounds, rosmarinic acid appeared to dominate in both subspecies. Lithospermic acid B, chlorogenic acid and isovitexin were present only in Greek oregano extracts. However, the total content of flavonoids was higher in common oregano extracts. The essential oil and extract of Greek oregano revealed visibly stronger antibacterial activity (expressed as MIC and MBC) than common oregano, whereas the antioxidant potential (determined by DPPH, ABTS and FRAP) of these extracts was almost equal for both subspecies. In the case of *Origanum* plants, the potential application of essential oils and extracts as antiseptic and antioxidant agents in the food industry should be preceded by subspecies identification followed by recognition of their chemotype concerning both terpene and phenolics composition.

## 1. Introduction

In the last decade, special attention has been paid to the search for natural compounds for preserving foods from spoilage microorganisms and oxidation [1,2]. This has become especially urgent due to the progressive withdrawal of commonly used synthetic antioxidants such as, e.g.: butylated hydroxytoluene (BHT), butylated hydroxyanisole (BAH) or propyl galate (PG) [3,4] and as a result of European Union (EU) law, prohibiting the application of antibiotic growth promoters in food production. The common use of in-feed antibiotics in large-scale livestock rearing has contributed to the increase in the resistance among human pathogens. Thus, since 2006 in the EU such practices are no longer acceptable in animal production [5,6]. This situation has caused the need to screen natural substances able to control growth of pathogens and prevent undesirable oxidation reactions in food products. Among foodborne bacteria, several species have gained a particular attention due to their high pathogenicity. Microorganisms such as *Bacillus cereus*, *Salmonella enterica*, *Escherichia coli*, *Staphylococcus aureus*, and *Listeria monocytogenes* can infect and intoxicate humans, leading to serious diseases manifested as abdominal pain, nausea, vomiting and diarrhea [7]. Among these, *Listeria monocytogenes* seems to be the most dangerous since it causes high mortality, especially in the case of pregnant women, newborns, and the elderly [8]. Thus, the search for a new, natural substances being able to control foodborne pathogens is focused on the abovementioned group.

Up to now, several plant-derived secondary metabolites, mainly phenolics, have been described to reveal activities against these pathogens. Among them the most interesting are phenolic acids (e.g., caffeic acids derivatives), flavonoids (e.g., (−)-epicatechin, quercetin), and volatiles, including essential oils and their components, e.g.: thymol, carvacrol or eugenol [9]. The antibacterial activity of phenolic acids is well-documented, especially when regards to rosmarinic acid. This compound shows potential in the control of *Bacillus subtilis*, *Pseudomonas aeruginosa*, *E. coli, S. aureus*, *Shigella* spp., and *Enterobacter* [10,11,12]. It has an inhibitory impact on the proteins of the microbial surface recognizing adhesive matrix molecules, what leads to damage to the bacteria cell wall [13]. Moreover, the presence of a carboxylic acidic group enables ionisability and/or the formation of salts with mineral cations, what was also observed in the case of antibiotics such as, e.g. daptomycin [14]. The activity of other phenolic acids, including chlorogenic acid, is based on a similar mechanism of action [2,15]. In the group of phenolic compounds, flavonoids exhibit a strong antibacterial activity expressed in an inhibition of nucleic acid synthesis, inhibition of cytoplasmic membrane function and by disorders in the energy metabolism of bacterial cells [16,17,18]. Volatiles show well-known antibacterial potential, especially essential oils and their components, e.g. carvacrol and thymol. Due to their hydrophobic nature, they are able to cross the lipid bilayer of cell membrane causing a loss of its integrity and leakage of cellular material. Unlike many antibiotics, these compounds gain access to the periplasm of Gram-negative bacteria such as *E. coli* and *Salmonella typhimurium* through the porin proteins of the outer membrane, and finally damage it irreversibly [19,20]. It is worth noting that the precursor of carvacrol, *p*-cymene, is not an effective antibacterial agent when used alone. However, when combined with carvacrol, synergism has been observed. When *p*-cymene is incorporated into the lipid bilayer, the transport of carvacrol is visibly facilitated [1,21]. Thus, the works on antibacterial and antioxidant activity should focus not only on single compounds but especially on complex extracts or their fractions. 

The phenolic compounds listed above, especially phenolic monoterpenes (carvacrol and thymol) and caffeic acid derivatives (rosmarinic and chlorogenic acids) are known for their high antioxidant potential. Their chemical structure with the presence of phenyl groups makes it possible for them to act as hydrogen atom or electron donors [22,23,24,25,26,27].

Plants rich in the abovementioned compounds are therefore a promising source of natural antimicrobial and antioxidant agents. Among this group, the genus *Origanum* (Lamiaceae) has gained particular attention because of its interesting chemical composition reflected in a dominance of phenolic compounds in both the volatile and non-volatile fractions [28]. *O. vulgare,* with six subspecies identified within this taxon, is an aromatic perennial sub-shrub, characterized by a high intraspecific variability. Each subspecies is associated with a main chemotype created on the basis of the ‘sabinyl’ or ‘cymyl’ pathways of monoterpenes biosynthesis, where only the second one leads to the formation of phenolic monoterpens (thymol/carvacrol) [29,30,31]. Two *O. vulgare* subspecies are especially important from an economic viewpoint, namely *Origanum vulgare* L. subsp. *hirtum* (Link) Ietswaart and *Origanum vulgare* L. subsp. *vulgare*. The first one also called Greek oregano, originates from the Mediterranean region and is especially valued due to the presence of pure carvacrol chemotypes (up to 80% carvacrol in the essential oil) [32,33]. The second one (common oregano) occurs on the Northern and Central Europe area as the only representative of the genus [34]. Both are cultivated and used commercially in the pharmaceutical, food and cosmetic industries and are known not only for antioxidant and antimicrobial, but also diuretic, expectorant, stimulative, carminative, antispasmodic, and anticancer activities [35,36,37]. It is known that antioxidant and antimicrobial activity of oregano-derived products is associated with the predominance of carvacrol and/or thymol in its essential oil followed by rosmarinic acid and its derivatives within the non-volatile fraction [1,9,22,33,38,39,40]. Although there is a large literature database concerning this issue, some problems are apparent. Previous studies on *O. vulgare* subspecies are numerous, however they seem to be incomplete, since their biological activities have usually been determined separately from the chemical composition. Given the subtle morphological differences between *O. vulgare* subspecies, they are often confused, not discriminated and/or treated as a collective taxon. Taking into account that each subspecies is usually assigned to its corresponding main chemotype (conditioning its biological properties), such mistakes may lead to wrong conclusions, especially when a raw material’s activity and application are concerned [33]. Another problem is related with the extraordinary variability of *O. vulgare*, where each subspecies itself is able to form various morphologically undistinguishable chemotypes [41,42,43,44]. These altogether may cause evident difficulties in practice, since non-phenolic chemotypes will not display the expected biological activity. It seems that in order to counteract such problems, oregano herb standardization should include subspecies identification combined with chemotype recognition. 

The aim of the present study was to compare *O. vulgare* L. subsp. *hirtum* and *O. vulgare* L. subsp. *vulgare*, in respect of antioxidant and antibacterial activity of the corresponding essential oils and hydroethanolic extracts in relation with their chemical profile. 

## 2. Results and Discussion

### 2.1. Essential Oil Content and Composition

The content of essential oil in the Greek oregano herb was at a level of 2.87 g × 100 g^−1^ DW, while in common oregano it was distinctly lower and amounted to only 0.53 g × 100 g^−1^ DW (Table 1). These results are in a good agreement with the data of other authors [30,31,33,45,46,47,48]. It is known that the content of essential oil in *O. vulgare* subspecies strongly depends on its geographical origin. Greek oregano together with other southernmost subspecies (subsp. *glandulosum* and subsp. *gracile*) are considered to be rich in volatiles, whereas common oregano, similarly to the other subspecies originating from central part of Europe (subsp. *virens* and subsp. *viride*), is regarded as a rather poor source of these compounds [45].

In the present work, 22 compounds were identified in Greek oregano essential oil, comprising 98.71% of the total identified fraction. In common oregano, 29 compounds were identified, amounting to 99.19%. Monoterpenes, with a predominance of monoterpene hydrocarbons, were the fundamental components of both analyzed essential oils. In Greek oregano, γ-terpinene and *p*-cymene were present in the highest amounts within this group. A considerable content of phenolic monoterpenes (39.79%) with a clear domination of carvacrol was also noticed in the case of this subspecies (Table 1). Such data are closely related to the literature [33,42,46,47,48,49]. The chemical composition of analyzed Greek oregano essential oil allowed us to classify the oil as a mixed carvacrol/γ-terpinene/*p*-cymene chemotype. It is known that this subspecies creates various pure or mixed chemotypes, where the most common are those with carvacrol and/or thymol as dominant components, as well as γ-terpinene and/or *p*-cymene-rich ones. Regarding industrial applications, the pure carvacrol chemotype (with up to 80% of carvacrol) is considered as the most valuable due to proven biological activity of this phenolic monoterpene [35]. This is reflected in European Pharmacopeia recommendation, where the sum of thymol and carvacrol in Greek oregano essential oil should not be lower than 60% [50]. 

In common oregano essential oil, sabinene and *p*-cymene dominated within the monoterpene hydrocarbons group (16.41 and 10.45%, respectively). In contrast to Greek oregano, phenolics (carvacrol and thymol) were present in much smaller quantities (7.11%) in favor of the oxygenated monoterpenes (21.19%) where terpinen-4-ol represented up to 13.81%. Common oregano essential oil was rich in sesquiterpenes, especially β-caryophyllene (6.87%) and its oxide (3.39%) (Table 1). The predominance of the abovementioned compounds allows us to qualify the essential oil as a mixed sabinyl/cymyl type rich in terpinen-4-ol. According to the literature, sabinyl chemotypes are considered to be the most frequent within common oregano, while the presence of phenolic ones is rather rare [30,31]. Common oregano is characterized by higher levels of terpenic polymorphism than Greek oregano, since a lot of different chemotypes have been recognized within this subspecies, as follows: sabinene, *cis*-sabinene hydrate, terpinen 4-ol, *p*-cymene + β-caryophyllene, germacrene d + β-caryophyllene, etc. [41,43,52,53].

### 2.2. Phenolic Compounds Composition

Greek oregano and common oregano hydroethanolic extracts differed in the content of determined phenolic acids and flavonoids. Regarding phenolic acids, five compounds were identified, namely: protocatechuic, caffeic, chlorogenic, rosmarinic and lithospermic B acids. In both subspecies, rosmarinic acid was the dominant compound (10,809.37 mg × 100 g^−1^ in Greek oregano and 8260.68 mg × 100 g^−1^ in common oregano). In the case of Greek oregano, the high content of rosmarinic acid was followed by a considerable amount of lithospermic acid B (7065.67 mg × 100 g^−1^) and chlorogenic acid (56.81 mg × 100 g^−1^). Interestingly, both compounds were not found in common oregano. In general, protocatechiuc, caffeic and chlorogenic acids were detected in visibly lower quantities in comparison to rosmarinic and lithospermic B ones. The content of caffeic acid appeared to be similar both in Greek oregano and common oregano (98.55 and 91.98 mg × 100 g^−1^, respectively), whereas the content of protocatechuic acid was almost three times higher in common oregano extract when compared to Greek oregano (Table 2). The presence of the above listed compounds in *Origanum* subspecies and the domination of rosmarinic acid was reported earlier by other authors [44,54,55,56,57]. According to Węglarz et al. [55] common oregano contains also *p*-hydroxybenzoic, vanillic and *o*-coumaric acid, while Raduciene et al. [54] showed the presence of chlorogenic acid in this subspecies. The results obtained by Grevsen et al. [44] indicate that Greek oregano is a rich source not only of rosmarinic acid, but also of lithospermic acid B and its derivative (epi-lithospermic acid B), what is in an agreement with our work. It is worth noting that the level of rosmarinic acid is quite variable between *O. vulgare* chemotypes [58]. For instance, in research performed by Lukas et al. [30], the content of this compound varied from 0.6 to 37.2 mg × g^−1^ in common oregano populations growing wild in Austria. Such a wide range of content suggests that rosmarinic acid, like terpenes, may be used as a marker of intraspecific variability. Moreover, when given its biological activity, this acid in plant material appears to be desirable from a pharmacological point of view [59,60].

In our study, six compounds were identified within the flavonoids group, as follows: luteolin 7-*O*-glucoside, apigenin 7-*O*-glucoside, naryngenin, isovitexin, (+)-catechin and (−)-epicatechin. Both, in Greek oregano and common oregano extracts, luteolin 7-*O*-glucoside and naryngenin were the dominant species. However, common oregano was characterized by a distinctly higher content of these substances. In turn, Greek oregano extracts were richer in (−)-epicatechin (Table 2). The content of apigenin 7-*O*-glucoside and (+)-catechin was similar in the extracts of both subspecies, whereas isovitexin was present only in Greek oregano. The obtained results correspond well with the literature data where the domination of luteolin, apigenin and naryngenin derivatives in *Origanum* subspecies was reported [38,44,54,55,56,57,61]. In Greek and common oregano, other non-methylated flavonones were noticed to be present in high amounts as well, namely eridictyol and its 6,8-di-C-glucoside [44,54,61]. According to Skoula et al. [61] many free flavones, flavonols, flavanones and dihydroflavonols have been identified within this taxon. Gonzalez et al. [56] claimed that the profile of phenolic compounds may be considered as a specific fingerprint useful in the case of authentication and/or discrimination of various *Origanum* subspecies.

### 2.3. Antioxidant Activity

The obtained results show that the antioxidant potential both of Greek oregano and common oregano essential oils and hydroethanolic extracts is similar (Table 3). The activity of essential oil derived from Origanum plants was reporter earlier by other authors [1,9,62,63]. According to Kulisic et al. [9], Greek oregano essential oil reveals remarkable antioxidant potential, comparable with α-tocopherol and the synthetic antioxidant butylated hydroxytoluene (BHT). Such an effect is considered to be related with the predominance of carvacrol and/or thymol in the essential oil. However, other monoterpenes, especially oxygenated ones, can also exhibit significant antioxidant activities [64]. When occurring together, these substances are suspected to act synergistically [9]. This may explain the similarity in the antioxidant power of Greek oregano and common oregano essential oils observed in the present study. Although common oregano essential oil contained small content of carvacrol and thymol, it was rich in other monoterpenes both oxygenated and hydrocarbons (52.99 and 21.19%, Table 1), probably responsible for its high antioxidant potential. These results are in a good agreement with data shown by Vazirian et al. [65], where common oregano essential oil, rich in oxygenated monoterpenes, revealed considerable antioxidant activity.

The antioxidant potential of selected extracts obtained from some *Origanum* subspecies has been already recognized [38,66,67,68]. Based on these data, it seems that the antioxidant activity is related to the solvent polarity, however, some inconsistency may be noticed there. For instance, Kaurinovic et al. [66] and Martins et al. [38] indicate on the aqueous extract as the most effective one, while Licina et al. [67] higlighted the ethanolic extract. It is worth noting that aqueous extracts are rarely used in the industry, in favor of hydroethanolic ones. The results obtained in the present study show that the antioxidant power of hydroethanolic extracts from Greek oregano and common oregano was at a similar level (Table 3). As it was mentioned earlier, the antioxidant activity of plants extracts is usually attributed to the presence of phenolic compounds, mainly phenolic acids and flavonoids. However, the chemical composition of phenolic’s fraction of investigated extracts was not identical (Table 2). Greek oregano extract includes a considerable content of lithospermic acid B, regarded as a strong antioxidant agent [69]. Nevertheless, common oregano extract contains four times more naryngenin, a flavonoid aglycon known for its high antioxidant power [70]. In general, common oregano extract is characterized by higher content of flavonoids, while Greek oregano contains more phenolic acids. This might provide to almost equal antioxidant activity of both investigated extracts

### 2.4. Antibacterial Activity

In order to express the antibacterial activity of investigated essential oils and hydroethanolic extracts against selected Gram-negative (*E. coli* ATCC 25922, *E. coli* O157:H7 ATCC 700728, *S. enteritidis* ATCC 13076) and Gram-positive (B. cereus ATCC 11778, *L. monocytogenes* ATCC 7644, *S. aureus* ATCC 25923) pathogenic microorganisms, MIC and MBC was determined. Essential oils and extracts were tested against standard reference strains of bacteria related to foodborn diseases. The study involved microdilution method, which is a well-suited technique for screening of numerous combinations of antimicrobial agents in relation to different bacteria. Moreover, this method is economical in terms of time and resources [71,72]. The value of bacteriostatic (MIC) and bactericidal (MBC) concentration of essential oils was presented in Table 4. 

In general, the activity of Greek oregano essential oil against the tested bacterial strains was at least three times higher when compared to the activity of common oregano essential oil. Here, Greek oregano essential oil effectively inhibited the growth of both Gram-negative and Gram-positive bacteria, with MIC values ranging from 0.25 to 1 mg × mL^−1^ and MBC values from 0.5 to 2 mg × mL^−1^. In turn, common oregano essential oil showed higher MIC values (2–8 mg × mL^−1^) and MBC (4–16 mg × mL^−1^), which is equivalent to less effective antibacterial activity compared of Greek oregano essential oil. Moreover, common oregano essential oil showed the weakest bacteriostatic and bactericidal activity on *S. aureus* ATCC 25923 in the examined range of concentrations (MIC = 32; MBC = 64 mg × mL^−1^). Taking into account MIC and MBC values, *E. coli* ATCC 25922 and O157:H7 ATCC 700728 appeared to be the most susceptible bacteria among tested strains. It seems that Gram-negative bacteria were more sensitive to the essential oils used in comparison to Gram-positive ones (Table 4). The observed antibacterial activity of essential oils may be related with their chemical composition. One mode of action of essential oil compounds (especially thymol and carvacrol) is the rapid depletion of the intracellular ATP pool through the reduction of ATP synthesis. As a result, the decrease of intracellular ATP content followed by proton motive force finally enhance the permeability of the membrane. The leakage of ions from the cell provide to membrane damage resulting in disturbances of the osmotic pressure of the cells [5,73,74,75]. In general, the antibacterial activity of *Origanum* genus essential oils is widely described in literature [33,38,75,76,77]. However, the comparison of the activity among different subspecies of the genus is rater scarce. Results obtained in the present study indicate on stronger antimicrobial activity of Greek oregano essential oil than common oregano. This may be explained by higher content of carvacrol (37.21%) followed by *p*-cymene (10.45%) in this essential oil (Table 1).

The antibacterial activity of hydroethanolic extracts from Greek oregano and common oregano against tested strains was also evaluated. Extracts inhibited the growth of all tested strains of bacteria, however, the MIC and MBC values were different. When regards Greek oregano extract, the MIC values were in range 2–4 mg × mL^−1^ and MBC from 4 to 8 mg × mL^−1^. Such results indicate on its effective antibacterial activity. In the case of common oregano extract, visibly higher concentration was applied in order to inhibit the growth of bacteria, especially Gram-negative strains. The weakest antibacterial activity was demonstrated by common oregano extract against *S. enteritidis* (MIC 64 mg × mL^−1^ and MBC > 64 mg × mL^−1^). The MIC and MBC values of those extracts against Gram-positive bacteria was in range of 4–8 mg × mL^−1^ (MIC) and 8–16 mg × mL^−1^ (MBC), except for *S. aureus* for which the MIC and MBC value was 32 mg × mL^−1^ and 64 mg × mL^−1^, respectively (Table 4). It was observed that Gram-negative bacteria were more resistant to investigated extracts when compared to Gram-positive strains. According to Martins et al. [38], hydroethanolic extracts obtained from *O. vulgare* inhibited the growth of *E. coli* and *P. aeruginosa strains*, while Licina et al. [67] demonstrated the antibacterial activity of such extracts against *Bacillus* and *S. aureus*. However, the abovementioned studies didn’t include the identification of *O. vulgare* subspecies. In general, the antibacterial activity of extracts investigated in our work may be associated with its chemical composition, especially when given phenolic acids fraction in which rosmarinic acid dominated. Visibly lower antibacterial activity of common oregano extract in comparison to Greek oregano, may be related with the absence of particular phenolic compounds such as lithospermic B and chlorogenic acids combined with the lower content of rosmarinic acid and (−)-epicatechin. These results correspond to those obtained by Sahin et al. [76] indicating that common oregano methanolic extract did not reveal antibacterial activity at all.

## 3. Materials and Methods

### 3.1. Plant Material

The objects of the study were two Origanum vulgare subspecies, i.e.: Greek oregano (*O. vulgare subsp. hirtum*; accession numbers: 406734) and common oregano (*O. vulgare subsp. vulgare*; accession number: 401204) cultivated at the experimental field of the Department of Vegetable and Medicinal Plants, Warsaw University of Life Sciences (WULS-SGGW) (5210180 N; 2105234 E). The seed material originated from Polish Genebank (National Centre for Plant Genetic Resources: Polish Genebank). The research was carried out on selected clones of the abovementioned accessions (one per each subspecies). The herb (upper, not woody parts of shoots) of both subspecies was harvested in June 2020, from 2-years-old plants, at the beginning of plant’s blooming. Plant material was dried at 35 °C and subjected to chemical analysis.

### 3.2. Preparation of Essential Oils

60 g of air-dried raw material was hydrodistillated 3 h using a Deryng-type apparatus [78]. Obtained essential oils were stored in dark vials at 4 °C. 

### 3.3. Preparation of Hydroethanolic Extracts

Air-dry, powdered raw material (10 g) was extracted with 100 mL of solvent (ethanol:water; 60:40, *v*/*v*) in Büchi Extraction System B-811 (Büchi Labortechnik AG, Flawil, Switzerland). Soxhlet hot extraction with twenty-five cycles was used. In order to obtain sufficient amount of extracts, the extraction was repeated 10 times. Obtained extracts were filtered, concentrated using a rotary evaporator Büchi R200 (Büchi Labortechnik AG), frozen in −80 °C for 5 days, and finally lyophilized (Labconco Freezone 2.5, Labconco, Kansas City, MO, USA). Dry extracts were powdered and stored in dark vials, at 4 °C. 

### 3.4. Chemical Analysis

The chemical composition of essential oil was determined using GC-MS and GC-FID (3.4.1), while the chemical composition of phenolic acids and flavonoids was analyzed by HPLC (see Section 3.4.2). All measurements were performed in triplicate.

#### 3.4.1. Analysis of Essential Oils by GC-MS and GC-FID

The analysis was performed by usage of an Agilent Technologies 7890A gas chromatograph combined with a flame ionization detector (FID) and MS Agilent Technologies 5975C Inert XL_MSD with Triple Axis Detector (Agilent Technologies, Wilmington, DE, USA). A polar capillary Omegawax^®^ column (30 m × 0.25 mm × 0.25 µm film thickness) was applied. Separation conditions were as follows: oven temperature isotherm at 60 °C for 2 min, then it was programmed from 60 °C to 220 °C at a rate of 4°C per min and held isothermal at 220 °C for 5 min. Separation conditions were previously described by Bączek et al. [79]. Essential oil compounds identification was based on comparison of mass spectra from the Mass Spectral Database, as following: NIST08, NIST27, NIST147, NIST11, Wiley7N2, and on comparison of retention indices (RI) relative to retention times of a series of n-hydrocarbons (C7–C30) with those reported in the literature [51]. 

#### 3.4.2. Analysis of Phenolic Acids and Flavonoids by HPLC

The parameters of chromatographic separation and integration as well as validation procedure was given earlier by Kosakowska et al. [60]. Validation parameters are presented in Table 5. The content of identified phenolic acids and flavonoids was calculated in mg × 100 g^−1^ of dry extract.

##### Validation

Standards were purchased from Sigma Life Science (Merck, Darmstadt, Germany) and ChromaDex^®^ (Irvine, CA, USA) and separately dissolved with MeOH in 25 ml volumetric flask according to the ChromaDex’s Tech Tip 0003: Reference Standard Recovery and Dilution and used as standard stock solutions [80]. Working solutions were prepared by diluting 10 µL and 100 µL of standard stock solutions with methanol in 10 mL volumetric flasks, 500 µL and 1000 µL in 5 mL volumetric flasks as well as 1000 µL in 2 mL volumetric flasks. The working solutions and undiluted stock solutions were injected (1 μL) on a column in six replicates (*n* = 6) using SIL-20AC HT. Six-point calibration curves were plotted according to the external standard method by correlating concentration with peak area. Curves parameters were calculated with Microsoft Excel 14 (Microsoft, Warsaw, Poland). Signal-to-noise ratio approach were used to determined LOD (S/N of 3:1) and LOQ (S/N of 10:1). The peak table and UV-spectra library (190–450 nm) of individual compounds were also created.

##### Separation Parameters

The work was performed using a Shimadzu Prominence chromatograph equipped with a SIL-20AC HT auto sampler, SPD-M20A photodiode array detector and LC solution 1.21 SP1 chromatography software (Shimadzu, Kyoto, Japan). Separations were achieved using a 100 mm × 4.60 mm, C18 reversed-phase column, 2.6 μm particles with solid core and porous outer layer (Kinetex™, Phenomenex, Torrance, CA, USA). Binary gradient of mobile phase A (deionised water adjusted to pH 2 with phosphoric acid) and B (ACN) was used as follows: 0 min—12.5% B; 4.0 min—23% B; 6.0 min—50% B; 6.01 min—12.5% B; 8 min—stop. The HPLC conditions were as follows: flow rate 1.5 ml×min^−1^, oven temperature 40 °C, injection volume 1 μL.

##### Integration Parameters

Peak identification was carried out by comparison of retention time as well UV-spectra with standards (Table 5).

### 3.5. Antioxidant Activity

#### 3.5.1. DPPH Scavenging Capacity Assay

The measurement of the DPPH (2,2-diphenylpicrylhydrazyl hydrate) radical scavenging activity was carried out according to Yen and Chen [81] with modifications concerning the time of reaction [82]. Dry hydroethanolic extract (0.25 mg) was dissolved in 1 mL of methanol (2×, 4×, 8×, 16× diluted solutions). Essential oils were also diluted in methanol (concentrations 0.20, 0.40, 0.80, 1.60, and 3.20 mg × mL^−1^) [83]. Then, 3 mL of methanol and 1 mL of DPPH methanolic solution (0.12 mg × mL^−1^) were added to 1 mL of the different concentrations of extracts and essential oils. Absorbance was measured after 10 min at 517 nm using a 1700 PharmaSpec UV/Vis spectrophotometer (Shimadzu, Warsaw, Poland). The antioxidant activity of extracts and essential oils was calculated as I = [(AB − AA)/AB] × 100, where I is DPPH inhibition (%); AB is the absorbance of a blank sample (t = 0 min); AA is the absorption of extract solution (t = 10 min). Trolox was used to estimate a standard curve. Results are expressed in μmol Trolox equivalents per g of extract and essential oil.

#### 3.5.2. ABTS Scavenging Capacity Assay

ABTS (2,2’-azino-bis(3-ethylbenzothiazoline-6-sulphonic acid radical caption) was measured according to the method described by Re et al. [84] and Arts et al. [85]. A stock solution was prepared by stirring 7 mM ABTS and 2.45 mM (final concentration) potassium persulfate in water and incubating at room temperature in the dark, for 16 h before use. The concentrated ABTS was diluted with phosphate buffered saline (PBS) to a final absorbance of 0.72 (±02) at 734 nm. Then 1 mL ABTS solution was added to 100 μL of extracts (2×, 4×, 8×, 16× diluted solutions) and 100 μL of essential oil (0.20, 0.40, 0.80, 1.60, and 3.20 mg/ml concentrations). Absorbance of ABTS was measured on a 1700 PharmaSpec UV/Vis spectrophotometer (Shimadzu) after 6 min incubation in dark, at 734 nm. The ability of the test sample to scavenge ABTS was compared to the Trolox standard. The solutions of Trolox were prepared in PBS, such that the final concentrations of the standards were 0.0, 1.25, 2.5, 5.0, 7.5, 10.0 mg × 100 mL^−1^. The percentage inhibition of ABTS of the test samples were calculated according to the following formula:

% inhibition = [(AB − AA)/AB] × 100

where AB is the absorbance of a blank sample and AA is the absorbance of the test sample. Results are expressed in mg Trolox equivalents per 100 mL of extract.

#### 3.5.3. Ferric Reducing Antioxidant Power Assay (FRAP)

A total of 0.25 mg of dry hydroethanolic extract was dissolved in 10 mL of methanol. The working reagent was prepared by mixing acetate buffer (300 mM, pH 3.6), a solution of 10 mM TPTZ (2,4,6-tris(2-pyridyl)-s-triazine) in 40 mM HCl, and 20 mM FeCl_3_·6H_2_O at 10:1:1 (*v*/*v*/*v*) ratio. A total of 100 μL of each properly diluted extract solutions were prepared in tubes with 3 mL of working reagent and shaken for 30 s. After 30 minutes of incubation in a water bath at 37 °C, the absorbance was measured at 593 nm (UV/Vis Shimadzu 1700 PharmaSpec, Shimadzu, Kyoto, Japan). The results were expressed as Trolox equivalent antioxidant capacity in μmol Trolox per g of extract and Fe^2+^ antioxidant capacity (Fe^2+^ μmol/g of extract) [86,87].

### 3.6. Antibacterial Analysis

#### 3.6.1. Target Bacteria and Inoculum Preparation

Reference strains originated from the American Type Culture Collection (ATCC, Manassas, VA, USA). In the study the following Gram-negative bacteria strains were used: *Escherichia coli* ATCC 25922, *Escherichia coli* O157:H7 ATCC 700728, *Salmonella enterica subsp. serovar Enteritidis* ATCC 13076, and Gram-positive bacteria: *Bacillus cereus* ATCC 11778, *Listeria monocytogenes* ATCC 7644 and *Staphylococcus aureus* ATCC 25923. 

All bacterial strains were kept frozen in glycerol stocks at −80 °C. Frozen bacterial strains were activate in tryptic soy broth (TSA, BTL, Warsaw, Poland) at 37 °C for 24 h in an aerobic condition. Next, the bacterial strains were transfer and diluted in sterile 0.85% saline solution (NaCl, POCH, Gliwice, Poland) to adjusted 0.5 on the McFarland turbidity scale (Densimat, bioMérieux, Marcy l’Etoile, France), which is equivalent to 1 × 10^8^ CFU × mL^−1^.

#### 3.6.2. Minimum Inhibitory Concentration (MIC) and Minimum Bactericidal Concentration (MBC)

Minimum inhibitory concentration (MIC) and minimum bactericidal concentration (MBC) of essential oils and hydroethanolic dry extracts were determined using microdilution assay [88]. At first, preparation of stock solution of essential oils and extracts were prepared. Essential oils were dissolve in sterile Müller-Hinton Broth (MHB) containing DMSO (40 µL) with 2% of Tween 80, and the final stock solutions were 64 mg × mL^−1^ Extracts were dissolved directly in sterile MHB and the final stock solutions were 64 mg × mL^−1^. 

Next, a series of twofold dilutions of tested essential oils and extracts in the concentration range from 64 to 0.125 mg × mL^−1^ were prepared in MHB, using sterile 96-well microtiter plates (Brand, Wertheim, Germany). To each well of plates 10 µL of bacterial inoculum was added and the final concentration of bacteria suspension was about 5 × 10^5^ CFU × mL^−1^. The well with medium and inoculum without essential oil or extract was also prepared and served as a negative control. In turn, wells with medium and essential oils or extracts without inoculum served as positive controls. The plates with bacteria were incubated at 37 °C for 24 h. The MIC value was defined as the lowest essential oil/extract concentration, in which no visual growth of bacteria was noted. To determine MBC value, 10 µL of broth was taken from the well showing no bacterial growth and spot-inoculated onto plates with Müller-Hinton Agar medium. The plates were incubated at 37 °C for 24h. The MBC was defined as the lowest concentration of essential oil or extract, which resulted in complete reduction of bacteria. Each experiment was performed in triplicate.

### 3.7. Statistical Analysis

Data were subjected to statistical analysis using Statistica 12 software (Cracow, Poland). The mean values were compared by using the one-way analysis of variance (ANOVA) and expressed as mean with standard deviation (±SD). The differences between individual means were signed as “*” in tables rows and considered to be significant at *p* < 0.05. Original results (replications) were given in Supplementary Materials (Table S1, Table S2). 

## 4. Conclusions

Greek oregano and common oregano differed in terms of chemical composition and biological activity of essential oils and hydroethanolic extracts. Due to the observed antioxidant potential, it seems that essential oils and extracts of both subspecies may be regarded as a promising source of natural antioxidants. However, considering the antibacterial properties, only Greek oregano should be taken into consideration, since such activity of common oregano essential oil and extracts was rather low. Obtained results are probably associated with various proportions of volatiles (i.a. carvacrol, thymol as well as other monoterpenes) and non-volatiles (i.e., rosmarinic and lithosperimic acid B) that may be responsible for limiting bacteria growth and inactivation of free radicals. To conclude, in the case of the *Origanum* plants, the potential application of its essential oils and extracts as antiseptic and/or antioxidant agents in the food industry, should be preceded by subspecies identification followed by recognition of their chemotype concerning both terpene and phenolics composition.

## Figures and Tables

**Table 1:** The total content (g × 100 g^−1^ DW) and gas chromatographic composition (% peak area) of essential oil samples.

No	Compound	RI^1^	RI^2^	Greek Oregano	Common Oregano
1	α-thujene	1024	1012–1039	5.00	3.24
2	α-pinene	1029	1008–1039	0.35	0.97
3	camphene	1074	1043–1086	0.98	0.06
4	β-pinene	1112	1085–1130	4.36	1.65
5	sabinene	1126	1098–1140	0.07	16.41
6	3-carene	1147	1122–1169	0.20	0.00
7	α-terpinene	1185	1154–1195	5.69	5.36
8	D-limonene	1204	1178–1219	0.38	1.08
9	α-phellandrene	1212	1148–1186	0.48	0.00
10	1.8-cineole	1214	1186–1231	0.00	3.57
11	(*E*)-2-hexenal	1216	1196–1238	0.13	0.00
12	*trans* β-ocimene	1236	1211–1251	0.00	2.23
13	γ-terpinene	1250	1222–1266	17.21	5.96
14	*p*-cymene	1275	1246–1291	11.13	10.45
15	*m*-cymene	1281	1244–1279	0.00	3.60
16	terpinolene	1284	1261–1300	0.39	1.98
17	3-octanol	1391	1372–1408	0.00	0.28
18	1-octen-3-ol	1446	1411–1465	1.44	1.28
19	linalool	1542	1507–1564	0.00	1.44
20	β-caryophyllene	1594	1570–1685	2.88	6.87
21	terpinen-4-ol	1597	1564–1630	3.62	13.81
22	*cis*-terpineol	1621	1616–1644	0.82	0.56
23	*trans*-terpineol	1674	1659–1724	0.30	1.76
24	α-humulene	1658	1637–1689	0.51	1.00
25	borneol	1684	1653–1728	2.98	0.00
26	β-bisabolene	1741	1698–1748	0.00	2.73
27	α-farnesen	1749	1714–1763	0.00	1.33
28	β-ionone	1846	1798–1892	0.00	0.05
29	caryophyllene oxide	1976	1936–2023	0.00	3.39
30	(−)-spathulenol	2125	2074–2150	0.00	0.66
31	thymol	2166	2100–2205	0.58	2.47
32	carvacrol	2213	2140–2246	37.21	4.64
33	α-cadinol	2229	2180–2255	0.00	0.36
	Total			98.71	99.19
	Monoterpene hydrocarbons			46.24	52.99
	Oxygenated monoterpenes			7.72	21.19
	Phenolic monoterpenes			39.79	7.11
	Sesquiterpene hydrocarbons			3.39	11.93
	Oxygenated sesquiterpenes			0.00	4.41
	Other compounds			1.57	1.56
	Essential oil content			2.87	0.53

^1^ RI—experimental retention index on a polar Omegawax^®^ column; ^2^ RI—range of retention indices on polar column reported by Babushok et al. [51].

**Table 2:** The chemical composition of phenolic acids and flavonoids in hydroethanolic extracts (mg × 100 g^−1^).

Compound	Greek Oregano	Common Oregano
**Phenolic acids**		
Protocatechuic acid	35.78 ± 0.24	99.66 ± 0.51 *
Caffeic acid	98.55 ± 1.28	91.98 ± 0.72
Chlorogenic acid	56.81 ± 0.90	-
Rosmarinic acid	10809.37 ± 552.32 *	8260.68 ± 69.61
Lithospermic acid B	7065.67 ± 39.94	-
**Total**	18066.18	8452.32
**Flavonoids**		
Luteolin 7-*O*-glucoside	611.68 ± 1.88	862.16 ± 6.49 *
Apigenin 7-*O*-glucoside	81.73 ± 1.18	75.77 ± 0.40
Naryngenin	451.46 ± 5.27	1196.02 ± 8.14 *
Isovitexin	273.44 ± 1.89	-
(+)-Catechin	14.68 ± 1.07	10.08 ± 0.30
(-)-Epicatechin	196.74 ± 0.31 *	51.47 ± 0.60
**Total**	1629.73	2195.5

* *p* < 0.05.

**Table 3:** Antioxidant activity of essential oils and hydroethanolic extracts (DPPH, ABTS, FRAP).

		Essential oils		Extracts	
Method		Greek Oregano	Common Oregano	Greek Oregano	Common Oregano
DPPH					
	(% RSC)	61.76 ± 0.06	62.01 ± 0.61	70.90 ± 0.03	69.83 ± 0.17
	(µmol Trolox/g)	220.29 ± 2.83	218.78 ± 2.68	252.10 ± 5.98	242.43 ± 1.62
ABTS					
	(% RSC)	68.34 ± 0.36	68.58 ± 0.12	76.25 ± 0.12	79.37 ± 0.60
	(µmol Trolox/g)	340.08 ± 3.10	342.96 ± 2.90	381.09 ± 2.40	397.22 ± 2.90
FRAP					
	(Fe ^2+^ µmol/g)	22.64 ± 3.20	24.12 ± 1.90	29.54 ± 1.95	31.10 ± 3.10
	(µmol Trolox/g)	223.57 ± 2.90	238.13 ± 1.70	287.80 ± 2.70	300.46 ± 2.60

* *p* < 0.05.; not significant.

**Table 4:** MIC and MBC values of essential oils and hydroethanolic extracts.

	MIC (MBC)			
	Essential Oils (mg × mL^−^^1^)		Extracts (mg × mL^−^^1^)	
Strain	Greek Oregano	Common Oregano	Greek Oregano	Common Oregano
**Gram-negative bacteria**				
*E. coli* ATCC 25922	0.25 (0.5)	2 (4)	4 (4)	64 (64)
*E. coli* O157: H7 ATCC 700728	0.25 (0.5)	2 (4)	4 (4)	64 (64)
*S*. *enteritidis* ATCC 13076	0.5 (1)	4 (8)	4 (4)	64 (>64)
**Gram-positive bacteria**				
*B. cereus* ATCC 11778	0.5 (1)	2 (4)	2 (4)	4 (8)
*L. monocytogenes* ATCC 7644	0.5 (2)	8 (16)	2 (4)	8 (16)
*S. aureus* ATCC 25923	1 (2)	32 (64)	4 (8)	32 (64)

**Table 5:** Validation parameters of the HPLC-DAD analysis (*n* = 6).

Compound	Precision Intraday (CV, %)	Rt (min)	λ (nm)	Precision Interday (CV, %)	Calibration Equation	*R*^2^ (*n* = 6)	Linear Range (mg × mL^−1^)	LOD (µg × L^−1^)	LOQ (µg × L^−1^)	Recovery (%)
Protocatechuic acid	0.30	1.42	254	0.80	y = 7102.9x + 43850.0	0.9996	0.38–380.00	6.69	22.28	96.4
Caffeic acid	1.00	2.10	325	1.72	y = 2592.9x + 379.6	0.9996	1.00–998.40	2.50	8.32	95.7
Chlorogenic acid	0.23	1.75	325	0.65	y = 1708.6x + 7483.2	0.9998	0.97–966.70	20.42	68.10	103.2
Rosmarinic acid	1.24	5.20	325	2.12	y = 2017.9x + 1100.4	0.9999	0.43–434.02	3.20	9.82	102.6
Lithospermic acid B	0.74	5.34	254	0.92	y = 1199.9x − 3549.8	0.9998	0.60–601.6	8.24	27.62	103.5
Luteolin 7-*O*-glucoside	0.36	4.10	335	2.67	y = 2022.2x −1149.4	0.9997	0.19–19.08	5.03	18.12	101.4
Apigenin 7-*O*-glucoside	1.14	4.85	335	2.45	y = 2673.3x + 4227.2	0.9998	0.20–195.40	7.52	25.87	97.8
Naryngenin	1.21	6.95	284	1.89	y = 1304.8x + 1983.8	0.9998	1.98–396.8	3.28	9.79	102.9
Isovitexin	1.00	2.15	335	1.33	y = 2096.1x − 904.8	0.9995	0.36–36.88	8.65	28.47	98.3
(+)-Catechin	0.34	1.61	203	1.21	y = 8216.4x − 6069.3	0.9998	0.95–950.00	10.90	36.40	102.7
(−)-Epicatechin	0.68	2.25	203	1.51	y = 7345.1x − 5643.8	0.9995	0.47–23.40	10.02	34.00	95.6

## Data Availability

The data presented in this study are available on request from the corresponding author.

## Supplementary Materials

The following are available online. Table S1. The chemical composition of phenolic acids and flavonoids in hydroethanolic extracts (mg × 100 g^−1^)—means with repetitions. Table S2. Antioxidant activity of essential oils and hydroethanolic extracts (DPPH, ABTS, FRAP)—means with repetitions.

## Sample Availability

Samples of compounds and plant materials used in the experiment are available from the authors.
