# Supplementary material for: Antioxidant and Antibacterial Activity of Essential Oils and Hydroethanolic Extracts of Greek Oregano (O. vulgare L. subsp. hirtum (Link) Ietswaart) and Common Oregano (O. vulgare L. subsp. vulgare)
Source: Molecules. 2021 Feb 13;26(4):988. doi: 10.3390/molecules26040988 (PMC7918425; doi:10.3390/molecules26040988)
Supplement: Supplementary file 1 [file molecules-26-00988-s001.pdf]

Supplementary materials concerning compounds identification

Analysis of essential oils by GC-MS and GC-FID

Separation conditions were at follows: oven temperature isotherm at 60°C for 2 min, then it was programmed from 60°C to 220°C at a rate of 4°C per min and held isothermal at 220°C for 5 min. Essential oil compounds identification was based on comparison of mass spectra from the Mass Spectral Database, as following: NIST08, NIST27, NIST147, NIST11, Wiley7N2, and on comparison of retention indices (RI) relative to retention times of a series of n-hydrocarbons (C7-C30) with those reported in literature [1].

Table S1. The comparison of experimental retention indices with those reported in literature.

No	Compound	RI[1]	RI[2]
1	α-thujene	1024	1012–1039
2	α-pinene	1029	1008–1039
3	camphene	1074	1043–1086
4	β-pinene	1112	1085–1130
5	sabinene	1126	1098–1140
6	3-carene	1147	1122–1169
7	α-terpinene	1185	1154–1195
8	D-limonene	1204	1178–1219
9	α-phellandrene	1212	1148–1186
10	1.8 cyneol	1214	1186–1231
11	2-hexenal, (E)	1216	1196–1238
12	*trans* β-ocimene	1236	1211–1251
13	γ-terpinene	1250	1222–1266
14	*p*-cymene	1275	1246–1291
15	*m*-cymene	1281	1244–1279
16	terpinolene	1284	1261–1300
17	3-octanol	1391	1372–1408
18	1-octen-3-ol	1446	1411–1465
19	linalool	1542	1507–1564
20	β-caryophyllene	1594	1570–1685
21	terpinen-4-ol	1597	1564–1630
22	*cis*-terpineol	1621	1616–1644
23	*trans*-terpineol	1674	1659–1724
24	α-humulene	1658	1637–1689
25	borneol	1684	1653–1728
26	β-bisabolene	1741	1698–1748
27	α-farnesen	1749	1714–1763
28	α-ionone	1846	1798–1892
29	caryophyllene oxide	1976	1936–2023
30	(-)-spathulenol	2125	2074–2150
31	thymol	2166	2100–2205
32	carvacrol	2213	2140–2246
33	α-cadinol	2229	2180–2255

[1] RI—experimental retention index on polar Omegawax® column

[2] RI—range of retention indices on polar column reported by Babushok et al. (2011) [1].

Analysis of phenolic acids and flavonoids by HPLC

Validation

The standards were purchased from Sigma Life Science (Merck, Darmstadt, Germany) and ChromaDex® (Irvine, USA) and separately dissolved with MeOH in 25 ml volumetric flask according to the ChromaDex's Tech Tip 0003: Reference Standard Recovery and Dilution and used as standard stock solutions [2]. Working solutions were prepared by diluting 10 µl and 100 µl of standard stock solutions with methanol in 10 ml volumetric flasks, 500 µl and 1000 µl in 5 ml volumetric flasks as well as 1000 µl in 2 ml volumetric flasks. The working solutions and undiluted stock solutions were injected (1 µl) on a column in six replicates (n=6) using SIL-20AC HT. Six-point calibration curves were plotted according to the external standard method by correlating concentration with peak area. Curves parameters were calculated with Microsoft Excel 14. Signal-to-noise ratio approach were used to determined LOD (S/N of 3:1) and LOQ (S/N of 10:1). The peak table and UV-spectra library (190-450 nm) of individual compounds were also created.

Parameters of separations

The work were performed using a Shimadzu Prominence chromatograph equipped with auto sampler SIL-20AC HT, photodiode array detector SPD-M20A and LCsolution 1.21 SP1 chromatography software (Shimadzu, Kyoto, Japan). Separations were achieved using a 100 mm × 4.60 mm, C18 reversed-phase column, 2.6 µm particles with solid core and porous outer layer (Kinetex™, Phenomenex, USA). Binary gradient of mobile phase A (deionised water adjusted to pH 2 with phosphoric acid) and B (ACN) was used as follows: 0 min – 12.5% B; 4.0 min – 23% B; 6.0 min – 50% B; 6.01 min – 12.5% B; 8 min – stop. The HPLC conditions were as follows: flow rate 1.5 ml×min^{-1}, oven temperature 40 °C, injection volume 1 µl.

Parameters of integration

Peak identification was carried out by comparison of retention time as well UV-spectra with standards (Table S2).

Table S2. Retention times and UV-spectra of standards.

Compound	Rt (min)	λ (nm)
Protocatechuic acid	1.42	254
Caffeic acid	2.10	325
Chlorogenic acid	1.75	325
Rosmarinic cid	5.20	325
Lithospermic acid B	5.34	254
Luteolin 7-*O*-glucoside	4.10	335
Apigenin 7-*O*-glucoside	4.85	335
Naryngenin	6.95	284
Isovitexin	2.15	335
(+)-Catechin	1.61	203
(-)-Epicatechin	2.25	203

References:

1. Babushok, V.I.; Linstrom, P.J.; Zenkevich, I.G. Retention Indices for Frequently Reported Compounds of Plant Essential Oils. *J. Phys. Chem. Ref. Data* **2011**, *40*.
2. https://www.chromadex.com/media/2126/techtip0003recoverydilutionprocedures_nl_pw.pdf
